# Pharmacist-led smoking cessation services in Ethiopia: Knowledge and skills gap analysis

**DOI:** 10.18332/tid/99573

**Published:** 2019-01-09

**Authors:** Daniel Asfaw Erku, Bisrat Hailemeskel, Adeladlew Kassie Netere, Sewunet Admasu Belachew

**Affiliations:** 1 Department of Clinical Pharmacy, School of Pharmacy, University of Gondar, Gondar, Ethiopia; 2 Department of Clinical and Administrative Pharmacy Sciences, College of Pharmacy, Howard University, Washington, United States

**Keywords:** smoking cessation, knowledge, practice, community pharmacy, Ethiopia

## Abstract

**INTRODUCTION:**

The present study’s objectives were: 1) assess the knowledge and attitude of pharmacists and pharmacy students regarding smoking/smoking cessation, and 2) document the extent of community pharmacists’ involvement in the provision of smoking cessation services in Ethiopia.

**METHODS:**

This study used cross-sectional and direct observation methods. A series of questionnaires were administered to final-year pharmacy students and practising pharmacists. Two scenarios simulating tobacco use in pregnancy and cardiovascular patients were selected and played by two well-trained simulated patients (SPs). Findings were analysed and presented using mean total scores, analysis of variances and independent sample t-test.

**RESULTS:**

A total of 410 participants (213 out of 238 pharmacy students, response rate 89.5%; 197 out of 361 pharmacists, response rate 54.6%) completed the survey. Both pharmacy students and practising pharmacists had positive attitudes towards smoking cessation, and both groups had similar mean knowledge scores. A total of 80 simulated visits were conducted. Recipients of training on smoking cessation had significantly higher mean knowledge and attitude scores compared with those who did not receive such training. The majority of the pharmacists demonstrated poor in history-taking practice, and seldom assessed the patients’ nicotine dependence level. Nicotine replacement therapies (NRTs) were supplied in only 10 of the visits and suggested, but not dispensed, in 35 of the visits. On the other hand, pharmacists in 59 visits counselled patients to visit addiction specialists and physicians.

**CONCLUSIONS:**

The present study revealed the presence of significant clinical knowledge gaps and inadequate skills among pharmacists regarding smoking cessation services. Educating pharmacists about smoking cessation support as part of their continuous professional development and providing a hands-on customised educational intervention, such as practice guidelines in the form of an Ask-Advise-Refer approach, about smoking cessation will be useful.

## INTRODUCTION

Tobacco use is one of the major preventable causes of death and disease^1^. There are about 1.1 billion smokers worldwide and nearly 80% of them currently reside in low and middle-income countries (LMICs)^2^. Sub-Saharan African countries are among regions that are now facing a substantial surge in tobacco use^3^. According to the 2016 Global Adult Tobacco Survey, 5% (3.4 million) of adults in Ethiopia use tobacco products and 6.5 million people are exposed to second-hand smoke^4,5^. According to findings from the Global Burden of Disease Study 2015, non-communicable diseases (NCDs) were the leading cause of age-standardised mortality rates in Ethiopia^6,7^. Even though there is no evidence on the magnitude of tobacco attributed mortality, an increase in smoking rate, coupled with a high prevalence of substance abuse, could be cited as a contributing factor to the increased mortality rates from NCDs^8^. Even though the WHO Framework Convention on Tobacco Control (FCTC) in 2014 and it’s national Tobacco Control Directive have been ratified, much of the work about enforcing anti-tobacco laws are still to be realised in light of the increasing interest of global tobacco companies to gain a share in the Ethiopian tobacco market^9^.

Ethiopia is experiencing a severe shortage of human resources in the health sector. In 2012, the Global Pharmacy Workforce Survey ranked Ethiopia 9th among countries with the least number of pharmacists^10^ with nearly 2000 actively practising pharmacists (2.38 pharmacists per 100000 population)^11^. Cognizant of the global shift in pharmaceutical practice, Ethiopia changed the undergraduate pharmacy curriculum from the traditional product-oriented pharmacy practice to a more patient-focused practice. The role of pharmacists in patient care and their involvement in public health priorities, such as smoking cessation services, is not yet realised despite an ongoing paradigm shift in pharmacy education.

Smoking cessation is one of the most important preventive health measures to reduce the risk of premature death and disease. In Ethiopia, smoking cessation support is not available in the majority of primary care facilities or the community^12^ and most of the cessation products and quitline services are not readily available except for nicotine patches and gums^13^. Advice provided by healthcare professionals can contribute to the success of efforts to quit smoking. Pharmacists, being the most accessible healthcare professionals among the community, offer a unique opportunity to deliver a more proactive smoking cessation service^14^. The role of pharmacists in dispensing smoking cessation products to a diverse group of customers trying to quit smoking also creates a tremendous opportunity to deliver sound smoking cessation support^15^. A recent systematic review and meta-analysis of published studies concluded that pharmacist-led behavioural support along with nicotine replacement therapies (NRTs) leads to a higher quit rate^16^. However, the role of pharmacists in developing countries, such as Ethiopia, is primarily confined to the traditional role of medication dispensing and their contribution to smoking cessation and other public health issues remains underutilised^17^. In Ethiopia, there is no pharmacy-delivered smoking cessation program^17^ and pharmacists are not trained to deliver behavioural support services to smokers. Taking the global evidence into consideration and due to lack of data in Ethiopia, the present study was conducted to: 1) assess the knowledge and attitude of pharmacists and pharmacy students regarding smoking/smoking cessation, and 2) document the extent of community pharmacists’ involvement in the provision of smoking cessation services.

## METHODS

### Study design

This study used cross-sectional and direct observation methods. Data were collected between 1 January and 30 November 2016. Ethical clearance was obtained from the ethical committee of the School of Pharmacy, the University of Gondar, before commencing the actual data collection. Participants were given a ‘consent to participate’ letter explaining the aim of the survey, the anonymity of the data to be collected and contact details of investigators.

### Cross-sectional survey

The first survey group were pharmacists practising in various pharmaceutical sectors in Ethiopia. All pharmacists who attended the annual meeting of the Ethiopian Pharmaceutical Association (EPA), a professional organization that embodies Ethiopian pharmacists working in some areas, were invited to participate. Questionnaires were distributed before the start of the meeting, and consenting pharmacists were requested to hand in the completed questionnaire forms after the meeting. In the second survey group, a convenience sample of all fifth-year (graduating class) pharmacy students enrolled at three public pharmacy schools in Ethiopia (University of Gondar, Wollo University and Jimma University) were included. In all survey groups, questionnaires were distributed, and consenting respondents were requested to return the completed questionnaires.

The data collection tool was adopted, with modification, from previously used instruments^14,18^ and prepared in English. This was translated to the Amharic language and back-translated to English to ensure that the translated version retained the intended meaning. The data collection instrument was pretested on 25 pharmacists, who were excluded from the final analysis, and appropriate modifications were made. The contents were then confirmed by the research team, including clinical pharmacists and public health experts. The questionnaire included two sections. Section one included items with ‘Yes’ or ‘No’ responses and short-answer questions requesting sociodemographic information. Part two included questions with a ‘True/False’ response and multiple choice questions covering epidemiologic questions and attitudes towards smoking and smoking cessation practices.

### Simulated patient study

The extent of community pharmacists’ involvement in providing smoking cessation support was evaluated using the direct observation method. The SP study was conducted in Addis Ababa, the capital city of Ethiopia. The city has more than 300 community drug retail outlets (CDROs) and is home for the majority of importers, wholesalers and pharmaceutical industries. The simulated visits targeted pharmacies and drug stores. Drug retail outlets in Ethiopia are classified based on the type of medications they are supposed to stock/dispense and qualifications of pharmacy staff. Pharmacies were defined as retail drug outlets having at least one licensed pharmacist with a minimum qualification of Bachelor of Pharmacy as pharmacy staff, whereas drug stores and retail outlets have at least one licensed pharmacy technician/druggist with a minimum requirement of a diploma in pharmacy as pharmacy staff. A sample of 81 CDROs was estimated for a 12% rate of counselling/smoking cessation support, a 95% confidence level and a 5% margin of error^19,20^. Using MS Excel random number generator (RAND), all CDROs were given a random number and stratified into a pharmacy or drug store. Overall, a total of 49 pharmacies and 32 drug stores were included using simple random sampling technique.

Two scenarios were developed. Scenario I, depicts a pregnant and heavy smoker woman (29 years old) who wants to quit smoking and is asking for help/advice. Scenario II, a young man (25 years old) is requesting a smoking cessation product and possible counselling for his father (61 years old), a known cardiovascular patient for the last 15 years. Two well-trained pharmacists acted as simulated patients (SPs). SPs were given detailed training and instruction regarding the scenarios and ways of approaching the pharmacists. Eventually, both SPs conducted a role play to make sure they will perform in an intended manner. The SPs accompanied each other every six visits to ensure the data recording and protocol were followed consistently. The data recording tool included a checklist of items classified into ‘preclinical actions’, including comprehensive patient history taking and assessment of dependence level (duration of smoking, number of cigarettes smoked per day, motivation to quit, products tried previously and their efficacy, current medical conditions, allergies and any current medications) and ‘clinical actions’, including pharmacological intervention (dose, when to take, duration, adverse effect and contraindications of the product dispensed, if any, or the product suggested) and non-pharmacological measures (lifestyle and/ or behavioral advice, follow-up monitoring and provision of any supporting material such as written information, and referals to addiction specialists and physicians). A score of ‘0’ was given for each item if the pharmacist did not correctly address the items, and a score of ‘1’ was given if the pharmacist correctly answered/addressed the items.

### Data analysis

The data collected from both the survey and SP visits were presented and analysed using descriptive statistics and mean total scores. Analysis of variance (ANOVA) and independent sample t-tests were employed to compare results between and within groups. A p-value of less than 0.05 was considered as statistically significant. All analyses were conducted using the Statistical Package for the Social Sciences (SPSS) software version 21.0 for Windows.

## RESULTS

### Results from cross-sectional survey

A total of 410 participants (213 out of 238 pharmacy students, response rate 89.5%; and 197 out of 361 pharmacists, response rate 54.6%) completed the survey. The general sociodemographic characteristics of respondents are shown in Table 1. The total knowledge and attitude scores of respondents are given in Table 2. Both practising pharmacists and pharmacy students had positive attitudes toward smoking cessation and its implications. The mean knowledge score was not significantly different between pharmacists and pharmacy students. However, ‘general’ knowledge scores such as the prevalence of smoking in Ethiopia and forms of tobacco smoking were higher in both students and pharmacists compared with more specific pharmacotherapeutic-related questions.

**Table 1:** Sociodemographic characteristics of respondents, Ethiopia, 2016 (N=410 )

*Characteristics*	*Students (N=213)*	*Pharmacists (N=197)*
**Mean age** (years ± SD)	24.3±1.4	29.8±7.6

**Table:** 

	*n (%)*	*n (%)*
**Gender**		
Male	69 (32.4)	110 (55.8)
Female	144 (67.6)	87 (44.2)
**Tobacco smoking status**		
Yes	14 (6.6)	10 (5.1)
No	199 (93.4)	187 (94.9)
**Training on tobacco use and quitting**		
Yes	40 (18.8)	51 (25.9)
No	173 (81.2)	146 (74.1)

**Table 2:** Total knowledge and attitude scores of students and pharmacists in Ethiopia, 2016

*Respondents*	*Students (% ± SD)*	*Pharmacists (% ± SD)*	*p*
Mean total knowledge score	63.4±9.8	65.2±9.9	0.47
Mean general knowledge score	67.8±13.2	66.3±9.8	0.33
Mean clinical/therapeutic score	41.7±16.9	37.9±15.4	0.19
Mean total attitude score	83.7±8.7	81.9±11.4	0.31

Moreover, when asked more specialised clinical knowledge questions such as tobacco-drug interactions and the importance of quitting in cardiovascular conditions, students scored a slightly higher level of correct responses compared to practising pharmacists. The exception to this, however, was a question regarding quitting during pregnancy where pharmacists scored higher in correct answers than students (72.6% vs 61%). Recipients of training on quitting tobacco use had significantly higher mean knowledge and attitude scores compared to those without training in smoking cessation.

### Results from direct observation study

Out of the selected 81 community pharmacies, one was found closed, and thus a total of 80 visits were conducted. The actual counselling practice of pharmacists on smoking/smoking cessation is summarised in Table 3. According to our findings, most pharmacists demonstrated very poor history-taking practice and seldom assessed the patients’ dependence level with only pharmacists in 9 of the visits asking about the number of cigarettes smoked per day and time to first cigarette. It was also observed that NRTs were supplied in only 10 of the visits (4 in Scenario I, 6 in Scenario II). Of these, full and appropriate information was provided along with the product in 7 of the visits. In 3 of the visits (2 in Scenario I, 1 Scenario II), however, a product was supplied based on the inappropriate advice of NRT being safe in the corresponding scenario. NRT product was suggested, but not dispensed in 35 of the visits (21 in Scenario I, 14 in Scenario II).

**Table 3:** Actual counselling practice of pharmacists on smoking/smoking cessation, results from the simulated visit in Ethiopia in 2016 (N=80 )

*Type of information*	*Total*	*Scenario I*	*Scenario II*
**Preclinical actions, mean total score (95 % CI)**		4.78 (3.81–4.93)	5.19 (4.61–5.71)
**History taking**			
Duration of smoking	12	6	6
Reasons for quitting	17	11	6
Products tried previously and their efficacy	7	2	5
Medical conditions	21	14	7
Allergies	4	1	3
Current medications	6	5	1
**Assessing dependence level**			
Time to first cigarette	9	3	6
Number of cigarettes per day	9	7	2
**Clinical actions, mean total score (95 % CI)**		6.82 (5.12–7.66)	7.44 (6.23–8.01)
**Pharmacological measures**			
Dispensed NRT products?	10	4	6
Exact dose and when to take dose	7	6	1
Instruction for use	7	5	2
Duration of therapy	7	4	3
Adverse effect and contra-indication	7	3	4
Recommends NRT products	35	21	14
**Non-pharmacological measures**			
Lifestyle and/or behavioral advice	65	28	37
Follow-up monitoring	11	7	4
Provide supporting materials*	31	8	23
Advice to visit addiction specialist	59	29	30

NRT: nicotine replacement therapy.

* Written information and websites.

On the other hand, most pharmacists (in 59 visits) counselled patients to visit addiction specialists and physicians. Overall, pharmacists were found to be less engaged in counselling practice and often referred patients to visit addiction specialist or general practitioners. Overall, pharmacists performed better in clinical actions, especially in non-pharmacological measures. Higher mean scores were also observed in the simulated patient representing a patient with cardiovascular diseases compared with the simulated pregnant patient.

## DISCUSSION

To the authors’ knowledge, this study is the first effort to evaluate the role of pharmacists in smoking cessation in Ethiopia. Two different groups were surveyed: practising pharmacists and final-year pharmacy students. We also conducted a simulated study to pinpoint the underlined gaps in providing smoking cessation services. According to our study, attitudes of both pharmacy students and practising pharmacists towards pharmacist-led smoking cessation were positive. While both groups had comparable knowledge scores, the general knowledge score was higher compared to the more specific pharmacotherapeutic-related and professional issues. This is not surprising since the prevalence of tobacco use is rising in the country and is becoming a major focus of public health campaigns and mass media in the last couple of years^21^. On the other hand, questions probing clinical knowledge such as tobacco-drug interactions and the importance of quitting in cardiovascular conditions were rated low, particularly among practising pharmacists, where they scored a slightly lower number of correct responses compared to students.

For pharmacists to be competent smoking cessation support providers, they should be equipped, preferably in their undergraduate courses, with the necessary knowledge and skills. Thus, private and public universities providing pharmacy education should integrate smoking cessation training within the required undergraduate or postgraduate curriculum. One such type of training could be to incorporate the 5-component smoking cessation treatment intervention model, known as the 5 As: 1) Ask about tobacco use, 2) Advise tobacco users to quit, 3) Assess readiness to quit, 4) Assist with quitting, and 5) Arrange follow-up care^22^.

In addition to the cross-sectional survey, SP visits were also employed to document the actual practice of pharmacists in smoking cessation. The scenarios were formulated to depict highly dependent smokers, with previously unsuccessful quit attempts and at high risk of adverse outcomes from smoking. It was apparent from the results of this study that pharmacists were poor at eliciting and taking a comprehensive patient history and seldom assessed the patients’ dependence level. This finding corroborates with other SP studies performed across the globe, all of which point towards a low level of involvement by pharmacists in taking medical history^23-25^. It was observed that rather than providing clinical measures, pharmacists often referred patients to see addiction specialists or physicians and offered non-pharmacological treatments.

Nevertheless, when a product was supplied, pharmacists provided sufficient information regarding dose, when to take a dose, duration of therapy, adverse effects and contraindications. The lack of training and/or experience in applying clinical knowledge in real life settings could be attributed to the poor performance of pharmacists in taking medical history^26^. Our study demonstrates the need for improving history-taking skills and improving awareness of current pharmacological and nonpharmacological options to quit smoking. Thus, educating pharmacists about smoking cessation support as part of their continuous professional development and providing a hands-on, customised educational intervention, such as a practice guideline about smoking cessation, would be useful.

This was a descriptive cross-sectional study based on an interviewer-administered questionnaire and a convenience sample. Thus, caution should be exercised when generalising to other parts of the country as the findings may have been subject to selection and social desirability bias. The data collection used for assessing the knowledge and attitude of pharmacy students and pharmacists had not been validated, and the reliability and psychometric properties were also not analysed. Moreover, as the scenarios enacted were too specific and conducted in only one city, it is difficult to generalise the results to other patient scenarios. Nonetheless, the findings from this study can serve as baseline data to develop, implement and evaluate pharmacy-led smoking cessation services in Ethiopia.

## CONCLUSIONS

Overall, the present study revealed the presence of significant clinical knowledge gaps among pharmacists concerning the current smoking cessation strategies. Overall skills of community pharmacists were also inadequate. Providing continuous clinical training and educational interventions are needed to mitigate the knowledge and skills gaps. One suggestion is offering a hands-on, customized educational intervention, such as practice guidelines in the form of an Ask-Advise-Refer approach, about smoking cessation in their practice areas. Large-scale studies exploring pharmacists’ involvement in smoking cessation support is recommended to identify practice barriers and to better inform academic and regulatory bodies.

## References

[1] Giovino GA, Mirza SA, Samet JM (2012). Tobacco use in 3 billion individuals from 16 countries: an analysis of nationally representative cross-sectional household surveys. The Lancet.

[2] World Health Organization (2018). Tobacco Fact sheet.

[3] Brathwaite R, Addo J, Smeeth L, Lock K (2015). A systematic review of tobacco smoking prevalence and description of tobacco control strategies in sub-Saharan African countries; 2007 to 2014. PLoS One.

[4] World Health Organization (2016). Ethiopia-GATS-2016-Executive Summary. 2016.

[5] Central Statistical Agency (2011). Ethiopia Demographic and Health Survey.

[6] Defar A, Getachew T, Teklie H (2017). Tobacco use and its predictors among Ethiopian adults: A further analysis of Ethiopian NCD STEPS survey-2015. Ethiopian Journal of Health Development.

[7] Misganaw A, Haregu TN, Deribe K (2017). National mortality burden due to communicable, non-communicable, and other diseases in Ethiopia, 1990–2015: findings from the Global Burden of Disease Study 2015. Population health metrics.

[8] Tesfaye F, Byass P, Wall S, Berhane Y, Bonita R (2008). Peer Reviewed: Association of Smoking and Khat (Catha edulis Forsk) Use With High Blood Pressure Among Adults in Addis Ababa, Ethiopia, 2006. Prev Chronic Dis.

[9] Ethiopian Food Medicine and Healthcare Administration and Control Authority (2015). Tobacco Control Directive.

[10] Federation of International Pharmacy (FIP) (2012). Global Pharmacy Workforce Report.

[11] Gebretekle GB, Fenta TG (2013). Assessment of pharmacists workforce in Ethiopia. Ethiopian Journal of Health Development.

[12] WHO (2017). WHO report on the global tobacco epidemic.

[13] FMHAHCA National Essential Medicine List Fifth Edition Addis.

[14] Saba M, Diep J, Saini B, Dhippayom T (2014). Meta-analysis of the effectiveness of smoking cessation interventions in community pharmacy. J Clin Pharm Ther.

[15] Philbrick AM, Newkirk EN, Farris KB, McDANEL DL, Horner KE (2009). Effect of a pharmacist managed smoking cessation clinic on quit rates. Pharm Pract.

[16] Brown TJ, Todd A, O’Malley C (2016). Community pharmacy-delivered interventions for public health priorities: a systematic review of interventions for alcohol reduction, smoking cessation and weight management, including meta-analysis for smoking cessation. BMJ open.

[17] Erku DA, Mersha AG (2017). Involvement of community pharmacists in public health priorities: A multi-center descriptive survey in Ethiopia. PLoS One.

[18] Goniewicz ML, Lingas EO, Czogala J, Koszowski B, Zielinska-Danch W, Sobczak A (2010). The role of pharmacists in smoking cessation in Poland. Eval Health Prof.

[19] Erku DA, Mekuria AB, Surur AS, Gebresillassie BM (2016). Extent of dispensing prescription-only medications without a prescription in community drug retail outlets in Addis Ababa, Ethiopia: a simulated-patient study. Drug, healthcare and patient safety.

[20] Nigussie WD (2014). Patient counselling at dispensing of medicines in health care facility outpatient pharmacies of Bahir Dar city, Northwest Ethiopia. Sci J Public Health.

[21] Tobacco Journal International Steady growth for Ethiopian tobacco sector.

[22] Tobacco TCPGT (2008). A clinical practice guideline for treating tobacco use and dependence: 2008 update: a US public health service report. Am J Prev Med.

[23] Chong WW, Aslani P, Chen TF (2013). Adherence to antidepressant medications: an evaluation of community pharmacists’ counseling practices. Patient preference and adherence.

[24] Chong WW, Aslani P, Chen TF (2014). Pharmacist–patient communication on use of antidepressants: a simulated patient study in community pharmacy. Research in Social and Administrative Pharmacy.

[25] Kippist C, Wong K, Bartlett D, Saini B (2011). How do pharmacists respond to complaints of acute insomnia? A simulated patient study. Int J Clin Pharm.

[26] Rosenthal M, Austin Z, Tsuyuki RT Are pharmacists the ultimate barrier to pharmacy practice change? Canadian Pharmacists Journal/Revue des Pharmaciens du Canada. 2010;.

